# Preparation and Characterization of Self-Microemulsifying Drug Delivery System of Olmesartan Medoxomil for Bioavailability Improvement

**DOI:** 10.1155/2013/728425

**Published:** 2012-09-24

**Authors:** Shailesh T. Prajapati, Harsh A. Joshi, Chhaganbhai N. Patel

**Affiliations:** ^1^Department of Pharmaceutics and Pharmaceutical Technology, Shri Sarvajanik Pharmacy College, Near Arvind Buag, Gujarat, Mehsana 384001, India; ^2^Department of Pharmaceutical Chemistry, Shri Sarvajanik Pharmacy College, Near Arvind Buag, Gujarat, Mehsana-384001, India

## Abstract

Olmesartan medoxomil (OLM) is an angiotensin II receptor blocker (ARB) antihypertensive agent administered orally that has absolute bioavailability of only 26% due to the poor aqueous solubility (7.75 *μ*g/ml). The aim of the present investigation was to develop a self-microemulsifying drug delivery system (SMEDDS) to enhance the oral absorption of OLM. The solubility of OLM in various oils, surfactants, and cosurfactants was determined. Pseudoternary phase diagrams were constructed using Acrysol EL 135, Tween 80, Transcutol P, and distilled water to identify the efficient self-microemulsification region. Prepared SMEDDS was further evaluated for its emulsification time, drug content, optical clarity, droplet size, zeta potential, *in vitro* dissolution, and *in vitro* and *ex vivo* drug diffusion study. The optimized formulation S2 contained OLM (20 mg), Tween 80 (33%v/v), Transcutol P (33%v/v), and Acrysol EL 135 (34%v/v) had shown the smallest particle size, maximum solubility, less emulsification time, good optical clarity, and *in vitro* release. The *in vitro* and *ex vivo* diffusion rate of the drug from the SMEDDS was significantly higher than that of the plain drug suspension. It was concluded that SMEDDS would be a promising drug delivery system for poorly water-soluble drugs by the oral route.

## 1. Introduction

Approximately, 40% of the new drug candidates in development today are water insoluble and associated with poor bioavailability. There were various formulation approaches reported to overcome these problems; these include the use of drug nanoparticles, solid dispersions, micronization, lipids, surfactants, complexation with cyclodextrin, and permeation enhancers [1]. Majority of these approaches have their limitations because of the need for specialized equipment, complicated manufacturing process, longer processing time, and regulatory complexity. Lipid-based formulation approaches, particularly the self-microemulsifying drug delivery system (SMEDDS), are well known for their potential as alternative approach for delivery of hydrophobic drugs [2], which are associated with poor water solubility and low oral bioavailability [3–5]. Lipid-based drug delivery system has gained considerable interest after the commercial success of Norvir (ritonavir), Fotovase (saquinavir), and Neoral (cyclosporine A).

Olmesartan medoxomil is a novel selective angiotensin II receptor blocker that is approved for the treatment of hypertension. It is a prodrug that is rapidly deesterified during absorption from the gastrointestinal tract to produce an active metabolite, olmesartan. However, the oral BA of olmesartan medoxomil was only 26% in healthy humans due to low solubility in water and unfavorable breakage of the ester drug to a poorly permeable parent molecule in the gastrointestinal fluids [6]. Efflux pumps in the gastrointestinal tract also interfere with drug absorption. Olmesartan dose-dependently reduces blood pressure through arterial vasodilatation and reduced sodium retention, as do other angiotensin receptor blockers [7].

SMEDDSs are isotropic and thermodynamically stable solutions consisting of an oil, surfactant, cosurfactant (CoS; or solubilizer), and drug mixtures that spontaneously form oil-in-water (o/w) microemulsions when mixed with water under gentle stirring. The motility of stomach and intestine provides the agitation required for self-emulsification *in vivo* [8]. SMEDDS spreads readily in the GI tract, and the digestive motility of the stomach and the intestine provides the agitation necessary for self-emulsification. This spontaneous formation of an emulsion in the gastrointestinal tract presents the drug in a solubilized form, and the small size of the formed droplet provides a large interfacial surface area for drug absorption [9]. Apart from solubilization, the presence of lipid in the formulation further helps improve bioavailability by affecting the drug absorption. Selection of a suitable self-emulsifying formulation depends upon the assessment of (1) the solubility of the drug in various components, (2) the efficient self-emulsifying region as obtained in the phase diagram, and (3) the droplet size distribution of the resultant emulsion following self-emulsification [10].

Thus, improving solubility and dissolution rate of olmesartan medoxomil can increase clinical efficacy or reduce the oral dosage required to achieve the same effect. Therefore, we use SMEDDS formulation with Acrysol EL135 as oil, Tween 80 as a surfactant, and Transcutol P as a co-surfactant to enhance the solubility and dissolution velocity of olmesartan medoxomil. The formulation was characterized for its ability to form microemulsions based on droplet size, zeta potential, and dissolution characteristics.

## 2. Materials and Methods

### 2.1. Materials

Olmesartan medoxomil was obtained as a gift sample from Alembic Pharma Ltd., Baroda, India. The following materials were gifted by Abitec Corp., USA, and were used as received: Capmul MCM (Glycerol monodicaprylate), Acconon C-80 (Polyoxyethylene 80 Coconut Glycerides), Captex 200 (Propylene Glycol Dicaprylocaprate), and Captex 355 (Glyceryl Tricaprylate). Transcutol P (highly purified diethylene glycol monoethyl ether), Plurol Oleique (polyglyceryl-3 dioleate), labrafil M 2125CS (linoleoyl macrogol-6 glycerides), Lauroglycol 90 (propylene glycol monolaurate) were received as gift sample from Gattefosse, France. Acrysol K 140 (polyoxyl 40 hydrogenated castor oil) and Acrysol El 135 (Polyoxyl 35 castor oil) were also gifted from Corel Pharma Chem., Ahmedabad, India. Tween 80 (polyoxyethylene sorbitan monooleate), Tween 60 (polyoxyethylene sorbitan monostearate), and propylene glycol were bought from Finar Chemical Limited, Ahmedabad, India. Polyethylene glycol 200 (PEG 200) and polyethylene glycol 400 (PEG 400) were bought from S. D. Fine Chemical Limited, Mumbai, India. Sunflower oil and castor oil were purchased from Gujarat Glycol Private Limited, Ankaleshwar, India, and Kush Proteins Private Limited, Anand, India. Double distilled water was used throughout the study. All other chemicals were of reagent grade.

### 2.2. Methods

#### 2.2.1. Selection of Self-Microemulsified Drug Delivery Systems Components

*Oil (Solubility Studies)*. Solubility of olmesartan medoxomil was determined in various modified oils, surfactants, and cosurfactants. Two mL of each component was taken in screw cap vials with known quantity (250 mg) of excess drug. A vortex mixer (Spinix, India) was used to facilitate the solubilization. Sealed vials were kept on isothermal mechanical shaker at 40 ± 2°C for 72 hours. After equilibrium, each test tube was centrifuged at 6000 rpm for 20 min using a centrifuge (R-8C, Remi, India). Supernatant was filtered through membrane filter using 0.45 *μ*m filter disk [11]. Filtered solution was appropriately diluted with methanol, and UV absorbance was measured at 257 nm. Concentration of dissolved drug was determined using standard equation. 

*Surfactant (Emulsification Study)*. Different surfactants were screened for its emulsification ability selected in oil phase. Surfactant selection was done on the basis of % transparency and ease of emulsification [11]. Briefly, 500 *μ*L of surfactant was added to 500 *μ*L of oil phase. The mixture was heated at 50°C for the homogenization of the components. Each mixture, 100 *μ*L, was then diluted with 50 mL distilled water in glass stopper conical flask. Ease of emulsification was judged by the number of flask inversions required to yield homogenous emulsion. The % transparency was evaluated at 650 nm by a double-beam UV spectrophotometer using distilled water as a blank. Emulsion was further observed visually for any turbidity or phase separation. 

*Co-Surfactant (Emulsification Study)*. The screening was done on the basis of % transparency and ease of emulsification. Mixtures of the co-surfactant, selected surfactant, and the selected oil were prepared and evaluated in similar fashion as described in the above section on surfactants [11].

#### 2.2.2. Drug-Excipients Compatibility Studies

**Figure 1:**
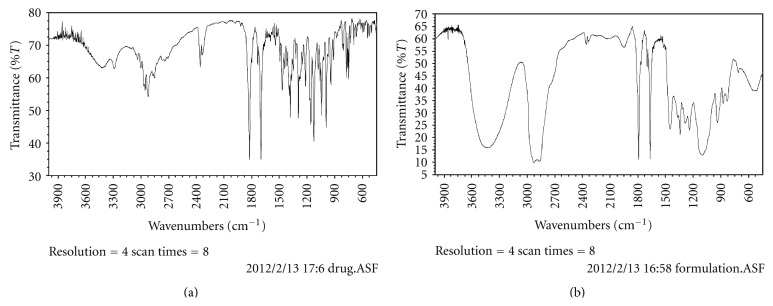
FTIR spectra of olmesartan medoxomil and formulation.

*FTIR Studies*. An FTIR-8400S spectrophotometer (Shimadzu, Japan) equipped with attenuated total reflectance (ATR) accessory was used to obtain the infrared spectra of drug in the isotropic mixtures of excipients. Analysis of pure drug, Acrysol EL135, Transcutol P, Tween 80, physical admixtures of the drug with the excipients (in 1 : 2 ratio), and their comelts (in 1 : 2 ratio) were carried out using diffuse reflectance spectroscopy (DRS)-FTIR with KBr disc. All the samples were dried under vacuum prior to obtaining any spectra in order to remove the influence of residual moisture. For each the spectrum, 8 scans were obtained at a resolution of 4 cm^−1^ from a frequency range of 4000–600 cm^−1^ as shown in Figure 1. 

*Pseudoternary Phase Diagram for SMEDDS Formulation*. Surfactant (Tween 80) and co-surfactant (Transcutol P) were mixed (Smix) in different volume ratios (1 : 1, 1.5 : 1, 2 : 1). For each phase diagram, oil (Acrysol EL 135) and specific surfactant/cosurfactant (Smix) ratio were mixed thoroughly in different volume ratios from 1 : 9 to 9 : 1 (1 : 9, 2 : 8, 3 : 7, 4 : 6, 5 : 5, 6 : 4, 7 : 3, 8 : 2, 9 : 1) in different glass vials. Pseudoternary phase diagrams were developed using the aqueous titration method [12]. Slow titration with the aqueous phase was performed for each combination of oil and Smix separately. The amount of aqueous phase added was varied to produce a water concentration in the range of 5% to 95% of total volume at around 5% time intervals. The calculation for the addition of aqueous phase was done by calculating the percentage of each component of the microemulsion present at each 5% addition. The beauty of this system is that the scale-up of the proportions is easy, as the system is thermodynamically stable. After each 5% addition of the aqueous phase to the oil : Smix mixture, visual observation was made and recorded. Through visual observation, the following categories were assigned: (1) transparent and easily flowable: oil/water microemulsions; (2) transparent gel: microemulsion gel; (3) milky or cloudy: emulsion; (4) milky gel: emulgel.

In a similar manner, calculations for the other ratios of oil and Smix were also done. For each Smix ratio, a separate phase diagram was constructed, and for each phase diagram visual observations were recorded. The pseudoternary phase diagram (Figure 3) was constructed using CHEMIX software based on the visual observations noted. In Figure 3, only microemulsion points are plotted (shaded area), so that there is no overcrowding of the phases in the diagram, as for formulation development only the microemulsion area is of interest. 

*Preparation of Self-Microemulsified Formulations*. OLM (20 mg) was added in accurately weighed amount of oil into a screw-capped glass vial and heated in a water bath at 40°C. The surfactant and co-surfactant were added to the oily mix using positive displacement pipette and stirred with magnetic bar. The formulation was further sonicated (Ultrasonic Cleaner EN-30-US, Electroquip, India) for 15 min and stored at room temperature until its use in subsequent studies [13]. Four SMEDDS formulations were prepared, and their self-emulsifying performance was compared. The composition of four formulations is shown in Table 1.

#### 2.2.3. Evaluation Parameters of OLM-Loaded SMEDDS

*Emulsification Time*. The emulsification time (the time for a preconcentrate to form a homogeneous mixture upon dilution) was monitored by visually observing the disappearance of SMEDDS and the final appearance of the microemulsion in triplicate. A visual test to assess the self-emulsification properties of SMEDDS formulation was performed by visual assessment as previously reported [14]. In this method, a predetermined volume of formulation (1 mL) was introduced into 300 mL of water in a glass beaker that was maintained at 37°C, and the contents mixed gently using a magnetic stirrer. The time to emulsify spontaneously and progress of emulsion droplets were observed. 

*Droplet Size Analysis*. Formulations (S1 to S4) each of 1 mL were diluted with 100 mL of water in a volumetric flask. The volumetric flask was inverted twice to ensure complete dispersion of the formulation. After ensuring complete dispersion of the formulation, the droplet size of resultant microemulsion was determined by photon correlation spectroscopy that analyzes the fluctuation in light scattering due to the Brownian motion of the droplets as function of time using a Zetasizer Nano Series (Malvern Instruments, DTS version 4.10, Serial number MAL 500999). Light scattering was monitored at 25°C at 90° angle. 

*Zeta Potential (ζ) Measurement*. Zeta potential of the formulations (S1 to S4) with oleylamine, without oleylamine was measured by using Malvern Zetasizer (Malvern Instruments, DTS version 4.10, Serial number MAL 500999) equipped with a 4.0 mW He-Ne red laser (633 nm). Zetasizer measures the potential that ranged from −120 to 120 V. For measurement of zeta potential 2 gm of each formulations were diluted with 100 mL water [15]. 

*In Vitro Dissolution Studies*. The *in vitro* drug release of OLM from the optimized SMEDDS was performed using USP dissolution Apparatus II (TDT-08L, Electrolab, Mumbai, India). Hard gelatin capsules, size “00” filled with preconcentrate (equivalent to 20 mg OLM) and pure drug (20 mg) separately, were put into each of 900 mL phosphate buffer pH 6.8, at 37 ± 0.5°C with a 50 rpm rotating speed. Samples (10 mL) were withdrawn at regular time intervals (5, 10, 15, 30, 45, and 60 min) and filtered using a 0.45 *μ*m filter. An equal volume of the dissolution medium was added to maintain the volume constant. The drug content of the samples was assayed using UV visible spectrophotometric method. All measurements were done in triplicate. 

*Determination of Drug Content*. OLM from SMEDDS formulation was extracted in methanol using sonication technique. The solutions were filtered, using Whatman filter paper. The methanolic extract was analyzed for the OLM content spectrophotometrically (UV-1800, Shimadzu, Japan) at 257 nm using standard curve. 

*Optical Clarity*. Each formulation (1 mL) was diluted with 100 mL of water in glass beaker. Absorbance of each dispersion was measured at 400 nm using a UV spectrophotometer immediately after microemulsions formation, and after 0 hrs, 6 hrs, and 24 hrs, respectively [16]. 

*In Vitro Drug Diffusion*. A 4-5 cm long portion of the dialysis tubing was made into dialysis sac by folding and tying up one end of the tubing with thread. It was then filled up with phosphate buffer saline pH 7.4 and examined for the leaks. The sac was then emptied and SMEDDS containing OLM equivalent to 5 mg diluted to 1 mL transferred into sac. One mL of the plain OLM (5 mg) suspension was accurately transferred into separate sac. The sacs were again examined for leak and then suspended in the glass beaker containing 50 mL phosphate buffer saline, which become the receptor compartment. At predetermined time intervals, 5 mL samples were withdrawn from the receptor compartment and analyzed by using UV spectrophotometer [17]. Fresh buffer was used to replenish the receptor compartment at each time point. The samples were withdrawn up to 8 hrs. The diffusion studies were done in triplicate.

#### 2.2.4. *Ex Vivo* Intestinal Permeability Study

All experiments and protocols described in this study were approved by the Institutional Animal Ethics Committee (IAEC), and all experiments were conducted as per the norms of the Committee for the Purpose of Control and Supervision of Experiments on Animals (CPCSEA), Ministry of Social Justice and Empowerment, Government of India. Male Wister rats (250–300 g) were sacrificed by CO_2_ inhalation method. Intestine was isolated and cleaned properly. One milliliter of the OLM SMEDDS and plain drug suspension sample (5 mg/mL) was filled into the intestine which was tied at both ends. The tissue was placed in an organ bath with continuous aeration at 37°C. The receptor compartment (organ tube) was filled with 30 mL of phosphate-buffered saline pH 7.4 with 1% sodium lauryl sulphate. At predetermined time intervals, samples were withdrawn from the receptor compartment [17]. Fresh buffer was used to replenish the receptor compartment. The samples were analyzed spectrophotometrically for the content of OLM. The percent diffusion was calculated and plotted against time. The study was also repeated with plain OLM suspension, and the results were compared. All the experiments were performed in triplicate.

## 3. Results and Discussions

### 3.1. Solubility Study (Screening of Oil)

Solubility studies were aimed at identifying a suitable oily phase for the development of the OLM SMEDDS. Identifying the suitable oil having the maximal solubilizing potential for the drug under investigation is very important to achieve optimum drug loading [18]. The solubility of OLM in various oils and surfactants is presented in Table 2. Among the various oily phases that were screened, Acrysol EL 135 could solubilize maximum amount of OLM (72.4 ± 2.31 mg/mL). The selection of the surfactant or cosurfactant in the further study was governed by the emulsification efficiency rather than the ability to solubilize OLM.

### 3.2. Preliminary Screening of Surfactants

Nonionic surfactants are generally considered less toxic than ionic surfactants. They are usually accepted for oral ingestion [19]. Results inferred that the oily phase Acrysol EL 135 exhibited the highest emulsification efficiency with Tween 80 for the homogenous emulsion formation. On the other hand, Acrysol EL 135 showed poor emulsification properties with other surfactants employed, requiring a higher number of flask inversions (Table 3). The aforementioned results suggested the use of Acrysol EL 135 as an oily phase with Tween 80 as a surfactant for further study.

### 3.3. Preliminary Screening of Cosurfactants

Addition of a co-surfactant to the surfactant-containing formulation was reported to improve dispersibility and drug absorption from the formulation [20]. As depicted in Table 3, Acrysol EL 135 exhibited good emulsification with Transcutol P showing the maximum transmittance (100%) followed by PG (99.4%) amongst all cosurfactants. Herein, the solubility of the drug in different cosurfactants may judge the final selection. Results of the solubility study demonstrated in Table 2 inferred a higher solubility in Transcutol P. It is worthy to note that all dispersions exhibited an instantaneous emulsion formation with only five flask inversions (Table 3). This could contend the importance of co-surfactant addition to the surfactant-containing dispersions.

### 3.4. Construction of Pseudoternary Phase Diagrams

**Figure 2:**
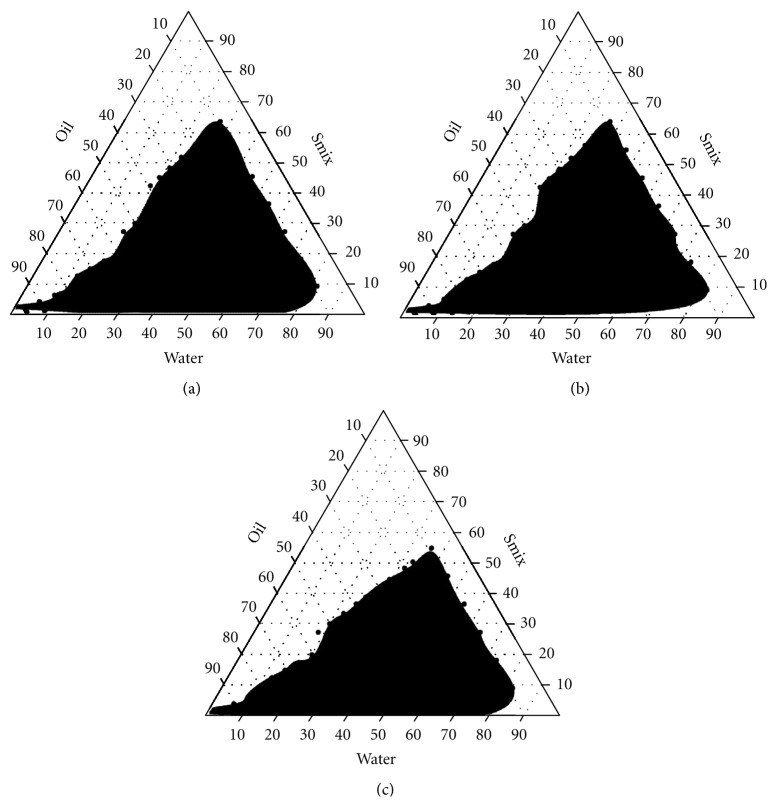
(a) Surfactant/co-surfactant 1 : 1, (b) surfactant/co-surfactant 1.5 : 1, and (c) surfactant/co-surfactant 2 : 1. Pseudoternary phase diagram of the system, Acrysol EL 135, Tween 80: Transcutol P, and water.

**Figure 3:**
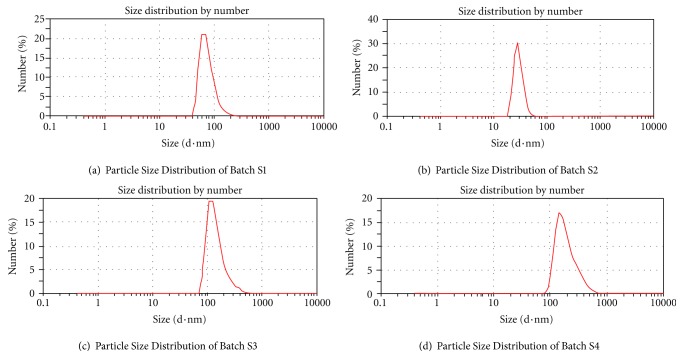
Particle size distribution of batches S1 to S4.

Pseudoternary phase diagrams were constructed in the absence of OLM to identify the self-emulsifying regions and to optimize the concentration of oil, surfactant, and co-surfactant in the SMEDDS formulations. A series of the SMEDDSs were prepared, and their self-emulsifying properties were observed visually. The phase diagrams were constructed at surfactant/co-surfactant ratios of 1 : 1, 1.5 : 1, and 2 : 1 (v/v). Phase diagram of different surfactant and co-surfactant ratio is shown in Figure 2. The gel-like region was found to become large with the increasing concentration of Tween 80, while the self-microemulsifying region decreased. The maximum self-microemulsifying region had to be at a ratio of 1 : 1. However, the drug precipitation was observed after several hours at ratios of 1.5 : 1 and 2 : 1. Cosurfactants are beneficial to form a microemulsion at a proper concentration range. However, an excessive amount of the co-surfactant will cause the system to become less stable for its intrinsic high aqueous solubility and lead to the droplet size increasing as a result of the expanding interfacial film [21]. Hence, the optimal ratio of surfactant to co-surfactant was selected to be 1 : 1 as shown in Figure 3.

Based on above results, a three-component SMEDDS formulation was established containing 34% Acrysol EL 135 as oil (on the basis of the solubility study and required target amount of OLM, 20 mg), 33% Tween 80 as the surfactant, and 33% Transcutol P as the co-surfactant (on the basis of phase diagrams). Four SMEDDS formulations were prepared, and their self-emulsifying performance was compared.

### 3.5. Evaluation of the SMEDDS

In the self-emulsifying systems, the free energy required to form an emulsion was very low, thereby allowing a spontaneous formation of an interface between the oil droplets and water. Moreover, since the drug released will be in nanosize, it will increase the effective surface area for dissolution.

#### 3.5.1. Emulsification Time

The efficiency of self-emulsification could be estimated primarily by determining the rate of emulsification which is an important index for the assessment of the efficiency of emulsification, that is, the SMEDDS should disperse completely and quickly when subjected to aqueous dilution under mild agitation. The emulsification time of these formulations was in the range of 15 to 35 sec (Table 4).

#### 3.5.2. Droplet Size and Zeta Potential Determination

The droplet size of the emulsion is a crucial factor in self-emulsification performance because it determines the rate and extent of drug release as well as absorption [22]. As depicted in Table 4, average droplet size was found in water, which ranges from 50.22 to 200.49 nm indicating all the particles were in the nanometer range. The result shows that the higher Smix proportion led to decrease in mean droplet size. The smallest particles were observed for formulation S2 (50.22 ± 3.42, mean ± SD, *n* = 3), and largest droplets were obtained for formulation S4 (Figure 3).

Zeta potential can be defined as the difference in potential between surface of the tightly bound layer (shear plane) and the electroneutral region of an emulsion. It has got practical application in the stability of emulsion since *ζ*-potential governs the degree of repulsion between adjacent, similarly charged, dispersed droplets. If the *ζ*-potential is reduced below a certain value (which depends on a particular system being used), the attractive forces exceed the repulsive forces, and the particles come together leading to flocculation. The zeta potential of the formulations were from −3.9 ± 0.42 to −23.89 ± 0.14 (mean ± SD, *n* = 3) as given in Table 4. In general, the zeta potential value of ±30 mV is sufficient for the stability of a microemulsion. All formulations comply with the requirement of the zeta potential for stability.

#### 3.5.3. *In Vitro* Drug Release

**Figure 4:**
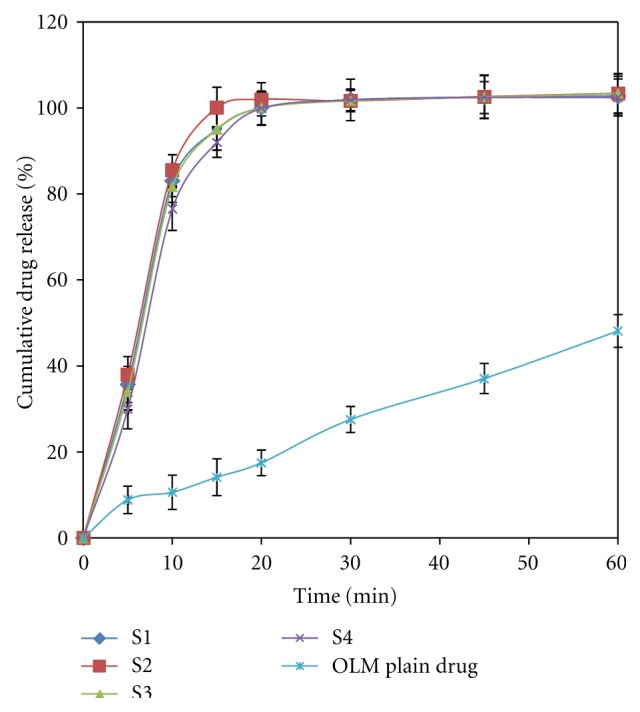
Dissolution profile of SMEDDS formulations and plain drug.

Dissolution studies were performed for the SMEDDS formulations in phosphate buffer pH 6.8, and the results were compared with the pure drug (Figure 4). There is no any significant difference in dissolution of four SMEDDS formulations. As the emulsification time is below 35 s, about 100% of the drug is released within 15 min in case of SMEDDS, while plain drug showed only 14.1% dissolution at the end of 15 min. The dissolution studies were conducted for 1 hr to observe the variation or occurrence of precipitation over a time. The *in vitro* dissolution studies indicate that formulation of OLM in the form of SMEDDS formulation enhances the dissolution properties.

#### 3.5.4. Drug Content

Irrespective of difference in composition, the drug content of formulations S1 to S7 was found in range of 99.35–101.79% (Table 4).

#### 3.5.5. Optical Clarity

Optical clarity measured by directly taking the absorbance of the diluted SMEDDS is a measure of droplet stability. The result indicates that formulation S2, and S3 were well stable till 24 hrs as their absorbance values did not change at the end of 24 hrs. Moderate changes in absorbance values were observed for formulation S1 at the end of 24 hrs. For formulation S4, a drastic change in absorbance values was observed indicating instability of droplets with time (Table 5).

#### 3.5.6. *In Vitro* Diffusion Studies

**Figure 5:**
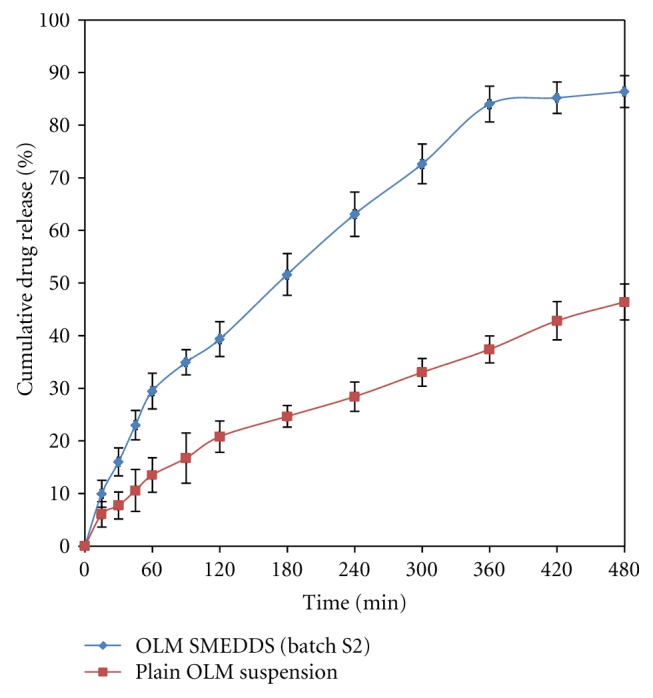
*In vitro *drug diffusion study of optimized SMEDDS (batch S2) formulation and plain drug.

*In vitro* diffusion of plain drug suspension and optimized SMEDDS is shown in Figure 5. The plain drug suspension was prepared by dispersing 20 mg of the drug in 5 mL of the distilled water. *In vitro* drug diffusion profiles are strong indicators of bioavailability. The amount of drug diffuse from SMEDDS was doubled than that of the plain OLM suspension. After 8 hours, 86.4% the drug was diffused from the SMEDDS, as compared with 46.39% diffused from the OLM suspension, indicating almost a twofold increase in the diffusion. The increased solubility and the dissolution rate are the main factors responsible for increased diffusion rates.

#### 3.5.7. *Ex Vivo* Intestinal Permeability Studies

**Figure 6:**
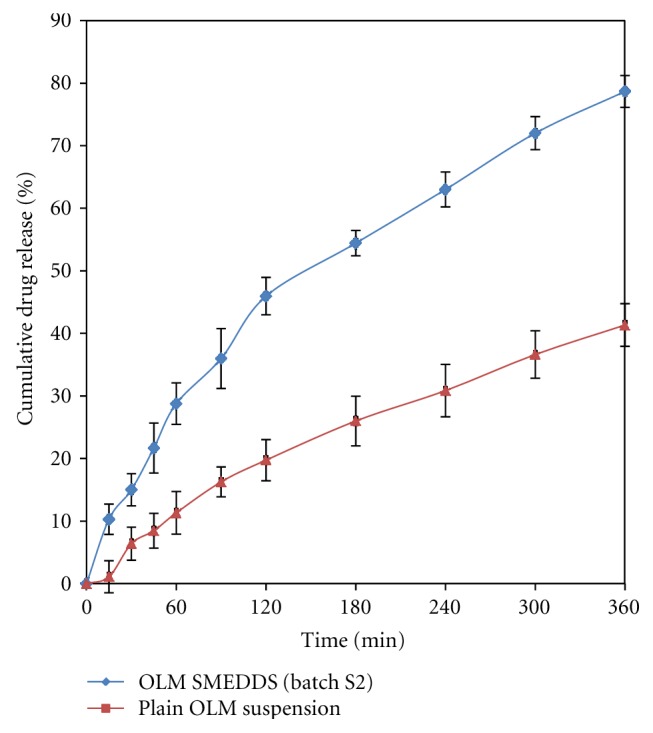
*Ex vivo *intestinal permeability study of optimized SMEDDS (batch S2) formulation and plain drug.

The results of the *ex vivo* intestinal permeability study are shown in Figure 6. After 6 h of diffusion, 78.7% of the drug was diffused from the SMEDDS, while from plain drug suspension the diffusion was found to be 41.3%. Thus, the amount of the drug diffused through the biological membrane has doubled when it is given in the form of a SMEDDS. The enhancement in diffusion is due to formation of microemulsion droplets in nanometer range and improved permeation of the OLM because of the presence of surfactant, which reduces the interfacial tension of formulation.

## 4. Conclusion

It was concluded that SMEDDS formulations containing olmesartan medoxomil significantly increase in the dissolution rate and *in vitro* diffusion rate compared to plain OLM suspension. The *ex vivo* intestinal permeability results showed that the drug diffusion across the intestinal membrane from the SMEDDS is significantly higher than the plain drug suspension. These observations lead us to the conclusion that SMEDDS seems to be a promising drug delivery system, which can provide an effective and practical solution to the problem of formulating drugs with low aqueous solubility and poor systemic bioavailability.

## Figures and Tables

**Table 1:** Selected formulations at a different % vol./vol. of oil, surfactant, and co-surfactant.

Formulation Code	Composition (% vol./vol.)		
	Acrysol El 135	Tween 80	Transcutol P
S1	30	35	35
S2	34	33	33
S3	36	32	32
S4	40	30	30

**Table 2:** Solubility of olmesartan medoxomil in few oils, surfactants, and cosurfactants.

Vehicle	Function in SMEDDS	Avg. solubility∗
		(mg/mL)
Capmul MCM	Oil	0.26 ± 0.01
Sunflower oil	Oil	0.358 ± 0.020
Castor oil	Oil	0.500 ± 0.045
Plurol Olieque	Oil	0.79 ± 0.23
Labraphil	Oil	0.76 ± 0.09
Lauroglycol	Surfactant	0.87 ± 0.02
Transcutol	Cosurfactant	36.4 ± 1.01
Captax200	Surfactant	0.29 ± 0.03
Captax355	Surfactant	0.69 ± 0.13
Propylene glycol	Cosurfactant	1.3 ± 0.77
Tween 80	Surfactant	73 ± 2.08
Tween 60	Surfactant	58 ± 1.89
Acconon C80	Cosurfactant	8.2 ± 0.31
Acrysol EL135	Oil	72.4 ± 2.31
PEG 200	Co-surfactant	1.52 ± 0.98
PEG 400	Co-surfactant	6.2 ± 0.95

∗Values are expressed as mean ± SD (*n* = 3).

**Table 3:** Emulsification efficiency of various surfactants, and cosurfactants.

Surfactants/co-surfactants	Transparency∗(%)	No. of inversions∗
Tween 80	99.8	5
Tween 20	98	15
Span 80	50.5	40
Transcutol P	100	5
Plurol Oleique	88.6	25
PEG 200	98.1	35
PEG 400	99.3	30
Propylene glycol	99.4	10
Capmul MCM C-8	80	40

∗Values are expressed as mean (*n* = 3).

**Table 4:** Evaluation parameters of formulations (S1 to S4).

Formulation code	Emulsification time (sec)∗	Particle size in water (nm)∗	Zeta potential (mV)∗	Drug content∗(%)
S1	17 ± 0.44	79.22 ± 2.32	−17.76 ± 0.35	99.94 ± 1.24
S2	20 ± 0.73	50.22 ± 3.42	−23.89 ± 0.14	99.85 ± 0.41
S3	25 ± 0.57	145.30 ± 1.90	−10.54 ± 0.23	99.33 ± 1.81
S4	35 ± 0.42	200.49 ± 3.08	−3.9 ± 0.42	101.79 ± 0.098

∗Values are expressed as mean ± SD (*n* = 3).

**Table 5:** Variation in optical clarity with time in water.

Formulation code	Absorbance at 400 nm∗		
	0 hrs	6 hrs	24 hrs
S1	3.2290 ± 0.0012	3.2000 ± 0.0026	3.0897 ± 0.0024
S2	0.0467 ± 0.0021	0.0472 ± 0.001	0.0479 ± 0.0001
S3	0.0570 ± 0.0011	0.0650 ± 0.0003	0.0671 ± 0.0022
S4	0.2990 ± 0.0013	0.3200 ± 0.0036	0.3506 ± 0.0026

∗Values are expressed as mean ± SD, *n* = 3.
